# Targeting of the class II transactivator attenuates inflammation and neurodegeneration in an alpha-synuclein model of Parkinson’s disease

**DOI:** 10.1186/s12974-018-1286-2

**Published:** 2018-08-30

**Authors:** Gregory P. Williams, Aubrey M. Schonhoff, Asta Jurkuvenaite, Aaron D. Thome, David G. Standaert, Ashley S. Harms

**Affiliations:** 1 Center for Neurodegeneration and Experimental Therapeutics, Department of Neurology, The University of Alabama at Birmingham (UAB), 1719 6th Ave. South, CIRC 525, Birmingham, AL 35294-0021 USA; 2 Department of Neurology, Houston Methodist Hospital, Houston, TX 77030 USA

**Keywords:** Parkinson’s disease (PD), Class II transactivator (CIITA), α-Synuclein, Major histocompatibility complex II (MHCII), Neuroinflammation, Neurodegeneration, Microglia, T cells, Monocytes

## Abstract

**Background:**

Parkinson’s disease (PD) is characterized by intracellular alpha-synuclein (α-syn) inclusions, progressive death of dopaminergic neurons in the substantia nigra pars compacta (SNpc), and activation of the innate and adaptive immune systems. Disruption of immune signaling between the central nervous system (CNS) and periphery, such as through targeting the chemokine receptor type 2 (CCR2) or the major histocompatibility complex II (MHCII), is neuroprotective in rodent models of PD, suggesting a key role for innate and adaptive immunity in disease progression. The purpose of this study was to investigate whether genetic knockout or RNA silencing of the class II transactivator (CIITA), a transcriptional co-activator required for MHCII induction, is effective in reducing the neuroinflammation and neurodegeneration observed in an α-syn mouse model of PD.

**Methods:**

In vitro, we utilized microglia cultures from WT or CIITA −/− mice treated with α-syn fibrils to investigate inflammatory iNOS expression and antigen processing via immunocytochemistry (ICC). In vivo, an adeno-associated virus (AAV) was used to overexpress α-syn in WT and CIITA −/− mice as a model for PD. Concurrently with AAV-mediated overexpression of α-syn, WT mice received CIITA-targeted shRNAs packaged in lentiviral constructs. Immunohistochemistry and flow cytometry were used to assess inflammation and peripheral cell infiltration at 4 weeks post transduction, and unbiased stereology was used 6 months post transduction to assess neurodegeneration.

**Results:**

Using ICC and DQ-ovalbumin, we show that CIITA −/− microglial cultures failed to upregulate iNOS and MHCII expression, and had decreased antigen processing in response to α-syn fibrils when compared to WT microglia. In vivo, global knock-out of CIITA as well as local knockdown using lentiviral shRNAs targeting CIITA attenuated MHCII expression, peripheral immune cell infiltration, and α-syn*-*induced neurodegeneration.

**Conclusion:**

Our data provide evidence that CIITA is required for α-syn-induced MHCII induction and subsequent infiltration of peripheral immune cells in an α-syn mouse model of PD. Additionally, we demonstrate that CIITA in the CNS drives neuroinflammation and neurodegeneration. These data provide further support that the disruption or modulation of antigen processing and presentation via CIITA is a promising target for therapeutic development in preclinical animal models of PD.

**Electronic supplementary material:**

The online version of this article (10.1186/s12974-018-1286-2) contains supplementary material, which is available to authorized users.

## Background

Parkinson’s disease (PD) is the most common neurodegenerative movement disorder, characterized by the progressive loss of dopaminergic neurons in the substantia nigra pars compacta (SNpc). The pathological hallmark of the disease is intracellular aggregates composed of the protein alpha-synuclein (α-syn) in Lewy bodies and Lewy neurites [1]. α-syn is hypothesized to play a pathological role in both genetic and sporadic forms of PD, as gene mutations and duplications are associated with earlier-onset, familial forms of PD [2], and genome wide association studies (GWAS) have strongly linked polymorphisms in the α-syn gene, particularly those that enhance expression, with increased risk for sporadic PD [3–5].

Recent evidence supports the idea that the activation of the immune system is involved in the disease process of PD and may be a critical link between α-syn and neurodegeneration. There is evidence for activation of both innate and adaptive immune system mechanisms in PD: HLA-DR reactive microglia surround areas of α-syn pathology in postmortem brain tissue, CD4 and CD8 T cell infiltration into the brain are observed, and pro-inflammatory cytokines are increased in blood and cerebrospinal fluid (CSF) of PD patients [6–9]. Several GWAS’s have found that genetic polymorphisms in the HLA-DR (human leukocyte antigen, part of the major histocompatibility complex II) locus are associated with sporadic PD [10–12], implicating antigen presentation in disease pathogenesis. Most recently, CD4 and CD8 T cells isolated from PD patients have been found to be auto-reactive to α-syn peptides [13]. Taken together, several immune mechanisms are essential in the pathobiology of PD, including MHCII activation and the adaptive immune system, and are potential targets for therapeutic development.

Utilizing an adeno-associated viral model of PD where full-length human α-syn is overexpressed in the SNpc of mice, we have shown that MHCII knockout mice display less reactive microglia and are completely protected from dopaminergic neuron loss [14]. This viral overexpression model recapitulates many of the neuronal and immune phenotypes observed in human PD, including reactive microgliosis, T cell infiltration into the CNS, increased pro-inflammatory cytokines, phospho-ser129+ (p-Ser129+) α-syn inclusions, and dopaminergic cell death in the SNpc [14–16]. However, MHCII global knockout mice do not possess functional CD4 T cells [17, 18], which confounds the interpretation of whether just MHCII, CD4 T cells, or both are responsible for mediating the neurotoxicity observed in response to α-syn expression. Additionally, from a therapeutic standpoint, targeting MHCII proteins in human disease would be problematic due to the heterogeneity of the protein within human populations [19, 20]. One potential upstream target for therapeutic investigation is the class II major histocompatibility complex transactivator (CIITA), a transcriptional coactivator that is necessary for both constitutive and inducible MHCII gene expression on antigen presenting cells (APCs) [21, 22]. CIITA serves as a critical regulator in tailoring an MHCII-mediated immune response by APCs such as microglia and macrophages in the CNS [18, 21, 22]. We sought to determine whether targeting inducible MHCII expression on CNS microglia via CIITA would be a viable therapeutic target to curb inflammation and subsequent neurodegeneration in PD.

To test if CIITA is required for α-syn-mediated inflammation and neurodegeneration, we utilized both a genetic knockout approach as well as an siRNA approach to locally target CIITA-mediated MHCII expression in the midbrain of mice, allowing us to bypass developmental abnormalities observed in MHCII knockout mice, i.e., the complete deficiency of CD4+ T cells. Here, we show that genetic knockout as well as midbrain-targeted siRNA silencing of CIITA does indeed attenuate α-syn-induced neuroinflammation and neurodegeneration in a mouse model of PD. These data show that the CIITA-mediated induction of MHCII is critical for the CNS as well as the infiltration of peripheral immune cells in response to α-syn, and that specific targeting of CIITA-induced MHCII expression may be useful in modifying disease progression.

## Methods

### Animals

Male and female C57BL/6 (catalog #000664; Jackson Laboratories) and CIITA knockout mice on a C57BL/6 background (B6.129S2-Ciita^tm1Ccum/J^, catalog #003239; Jackson Laboratories) [18] were used for these studies. All animals were maintained on a congenic, homozygous background. All research conducted on animals was approved by the Institutional Animal Care and Use Committee at the University of Alabama at Birmingham.

### α-syn fibril preparation

Preparations of human α-syn preformed fibrils were produced and purified as previously described [23] and then stored at − 80 °C. To make the working solution of preformed fibrils, 20 μl of 5 mg/ml stock fibril solution was diluted into 980 μl of sterile PBS as described [16, 23]. The fibrils were sonicated using a Fisher Scientific Sonic Dismembrator Model 500 with a program consisting of sonication at 10% power for 30 s at intervals of 0.5 s on/off. The control used for the experiment was human α-syn monomer that is stored at − 80 °C to prevent aggregation and then maintained on ice until addition into media. Monomer was centrifuged at 4 °C at 30,000×*g* for 10 min, and supernatant was added to the cells.

### Primary microglia cultures

Primary murine microglia were isolated from postnatal day 0–2 pups according to previously published protocols [14] with a few modifications. Briefly, brains were isolated, meninges were removed, and were dissociated for 10 min at 37 °C with frequent agitation. Mixed glial populations were filtered through a 40-μm filter and plated in T75 flasks in DMEM/F12 supplemented with 20% heat inactivated fetal bovine serum (Sigma-Aldrich), 1% penicillin/streptomycin (Sigma-Aldrich), 1% l-glutamine (Sigma-Aldrich), and 10 ng/ml granulocyte monocyte colony stimulating factor (PeproTech) for 10–14 days. Microglia were isolated from the astrocyte bed by mechanical shaking at 195 rpm for 1 h at 37 °C and were counted and plated.

### Immunocytochemistry for iNOS and DQ-ovalbumin antigen processing

Wild type and CIITA −/− microglia were plated in chamber slides (Lab-Tek II Chamber Slides) at 100,000 cells per well. Before assays, microglia were allowed to settle onto chamber slide for 2 h, and then washed with fresh media. Sonicated, preformed α-syn fibrils (200 ng/ml) or α-syn monomer as control were added into media in the chambers for 2 h. For iNOS quantification experiments, microglial cells were stained with anti-iNOS (Abcam) antibodies as described previously [14]. For antigen processing experiments, cells were treated with DQ-ovalbumin (Invitrogen) for 1 h prior to fixation. Upon hydrolysis of the DQ-ovalbumin, the FITC conjugated BSA protein becomes brightly fluorescent, and this fluorescence was quantified. All cells were fixed with 2% paraformaldehyde in 0.01 PBS, washed with PBS three times, and coverslipped. Imaging was performed using a Leica TCS-SP5 laser scanning confocal microscope. Four images per chamber well slide were captured, with 30–40 microglia per image. Each individual chamber was quantified with *n* = 4 per slide per treatment. Mean fluorescence of individual cell staining in each image was quantified using ImageJ software.

### Stereotaxic surgery

Male and female C57BL/6 (WT) and CIITA mice (8–12 weeks of age) were anesthetized with isoflurane and unilaterally (immunohistochemistry experiments) or bilaterally (flow cytometry experiments) injected with 2 μl of AAV2-SYN or AAV2-SYN together with 2 μl of lentiviral shRNAs (3.3 × 10^8^ IU/ml diluted in sterile PBS) into the right SNpc. Coordinates were anterior–posterior − 3.2 mm from bregma, mediolateral + 1.2 mm from midline, and dorsoventral − 4.6 mm from dura.

### AAV2 virus and siRNA lentiviral construction

Construction and purification of the rAAV vectors rAAV-CBA-IRES-EGFP-WPRE (CIGW) and rAAV-CBΑ-SYNUCLEIN-IRES-EGFP-WPRE (CSIGW) have been described previously [24].

To specifically silence CIITA expression, we produced six different shRNAs (Dharmacon cat# RMM4532-EG12265 glycerol set) which were packaged and processed into a mature pFUGW lentiviral construct (Addgene, plasmid #14883) [25, 26]. In brief, the preparation of the viral particles was done using cFUGW shRNAmir constructs co-transfected with packaging plasmids (pLP1, pLP2, pVSV-G (Invitrogen catalog #K4975-00) into HEK-293FT cells with FuGENE HD Transfection Reagent (Promega). Supernatant was collected 48 h and 72 h after transfection and concentrated by ultracentrifugation for 3 h at 100,000*g*, at 4 °C. The lentiviral particles were re-suspended in serum-free OPTIMEM and stored at − 80 °C. Viral titering was performed according to manufacturer’s protocol using HEK293T cells. Additionally, a control lentivirus was purchased with the same viral backbone (GIPZ Lentiviral shRNA vector, Dharmacon category #RHS4584) containing eGFP and titered to the same concentration as experimental lentiviruses.

### Immunohistochemistry

At 4 weeks and 6 months post viral transduction, mice were anesthetized, euthanized, and the brains were collected for processing as previously described. Briefly, animals were perfused with heparinized 0.01 M PBS followed by 4% paraformaldehyde, drop-fixed overnight, and transferred for cryoprotection to 30% sucrose in PBS. Brains were frozen on dry ice and coronal sections 40 μm thick were serially collected using a sliding microtome. Sections were stored in 50% glycerol in 0.01 M PBS at − 20 °C until used for staining.

For diaminobenzadine (DAB) staining, sections were washed with Tris-buffered saline (TBS and labeled as previously published [14, 16, 27]. Briefly, anti-MHCII (M5/114.15.2; eBiosciences), anti-alpha-synuclein (phospho S129) (EP1536Y, Abcam), or anti-TH (Millipore) antibodies were diluted in 1% normal serum in TBS-Triton (TBST) and incubated with sections overnight at 4 °C. Appropriate biotinylated secondary antibody (Vector Laboratories) was diluted in TBST + 1% serum and incubated for 2 h at room temperature. R.T.U. Vectastain ABC reagent (Vector Laboratories) and DAB kit (SK-4100; Vector Laboratories) were used according to the manufacturer’s instructions to develop HRP reactions. Co-labeling was achieved by using nickel DAB (Ni-DAB. Sections were mounted onto coated glass slides, dehydrated, and coverslipped using Permount mounting medium (Fisher).

### MHCII imaging and quantification

Images acquired on a Nikon Eclipse Ti-E. For MHCII DAB staining quantification, slides were blinded, scanned, and quantified by the mean gray value method via ImageJ (NIH). Briefly, three midbrain sections per animal encompassing the SNpc were chosen for quantification. The MHCII DAB mean gray value (average pixel value in a selected region) was calculated for both ipsilateral and contralateral sides (normalized to mean gray value of non-tissue slide background). MHCII fold induction was determined by dividing ipsilateral mean gray values by contralateral mean gray values and an average fold induction was determined per animal, *n* = 4–6 animals were quantified per treatment group.

For Additional file 1B, MHCII staining was scored using a numerical scale of 0 (no staining) to 4 (most intense staining) by a single observer blinded to the treatment paradigm, per previous publications [14, 16, 28].

### Unbiased stereology

TH neuron quantification was performed using unbiased stereology as previously published [14, 16, 24, 29]. In short, free floating sections (40 μm thickness, every sixth section throughout the SNpc) were immunostained for TH, blinded and analyzed on an Olympus BX51 with MicroBrightfield software. Five midbrain sections positive for TH, encompassing the rostrocaudal extent of the SNpc from each experimental group were quantified on both the injected ipsilateral and uninjected contralateral sides using the optical fractionator method and StereoInvestigator software. Neurons that stained positive for TH (DAB+ in the cell body with no staining in the nucleus) were counted on a 100 μm × 100 μm grid with a 50 μm × 50 μm counting frame and an optical dissector height of 22 μm. Variations in section thickness were accounted for by using weighted section thickness. The weighted section thickness was measured at every tenth sampling site and determined to be an average of 33 μm. A Gunderson coefficient of error < 0.10 was considered acceptable.

### Mononuclear cell isolation and flow cytometry

Mononuclear cells were isolated 4 weeks post-transduction from ventral midbrains with bilateral AAV injections, according to published protocols [27, 30]. Briefly, midbrains were digested with 1 mg/mL Collagenase IV (Sigma) and 20 μg/mL DNAse I (Sigma) diluted in RPMI 1640 with 10% heat inactivated fetal bovine serum, 1% L-glutamine (Sigma), and 1% Penicillin-Streptomycin (Sigma). Mononuclear cells were separated out using a 30/70% Percoll gradient, as previously described [30]. Isolated cells were blocked with anti-Fcy receptor (clone 2.4G2 BD Biosciences) then incubated with fluorescent-conjugated antibodies against CD45 (clone 30-F11, eBioscience), CD11b (clone M1/70, BioLegend), MHCII (M5/114.15.2, BioLegend), Ly6C (clone HK 1.4, BioLegend), CD4 (clone GK1.5, BioLegend), and CD8a (clone 53-6.7, BioLegend). A fixable viability dye was used to distinguish live cells from debris per manufacturer’s instructions (Fixable Near-IR LIVE/DEAD Stain Kit, Invitrogen). Samples were analyzed using an Attune Nxt flow cytometer (Thermo Fisher Scientific) and FlowJo software (Tree Star).

### Statistical analysis

For quantification of iNOS levels in primary microglia, mean fluorescence was compared in WT and CIITA −/− cells treated with either α-syn monomer or α-syn fibrils. A two-way ANOVA with Tukey’s multiple comparisons was performed, and mean ± SEM is displayed in graphs. To quantify DQ-ovalbumin, images were also analyzed for mean fluorescence. A two-way ANOVA with Bonferroni’s multiple comparisons was used to compare WT or CIITA −/− cells treated with either α-syn monomer or α-syn fibril, and mean ± SEM is displayed in graphs.

For DAB MHCII staining quantification, four to six animals were analyzed per group. An unpaired t-test (WT versus CIITA−/− experiments) or a one-way ANOVA with Dunnett’s test (Lentiviral silencing experiments) was used to compare groups. Graphs displayed the mean ± SEM.

For unbiased stereology, TH+ neurons in both contralateral and ipsilateral sides of 7–10 mice per group were counted. Ipsilateral, injected size was normalized to the contralateral, uninjected side as an internal control. This normalized value is presented as percent of contralateral side. For CIITA −/− versus WT comparisons, a two-way ANOVA with Tukey’s multiple comparisons was utilized. Mean ± SEM is displayed on graphs. For lentiviral silencing experiments, both contralateral and ipsilateral sides are presented for clarity. Grouped *t* tests were performed within treatment groups to demonstrate that treatments prevented neurodegeneration of the ipsilateral side compared to the contralateral side. Graphs display the mean ± SEM.

Flow cytometry experiments utilized four independent samples per group, with two ventral midbrains pooled per sample. Therefore, each experiment used a total of 24 mice. Data was analyzed using a one-way ANOVA with Tukey’s multiple comparisons. The mean ± SEM are plotted on graphs. For all statistical analyses in this paper: *p < 0.05, **p < 0.005, ***p < 0.0005, ****p < 0.0001.

## Results

### Genetic knock-out of CIITA reduces α-syn-induced inflammation, antigen processing, and presentation in vitro

**Fig. 1:**
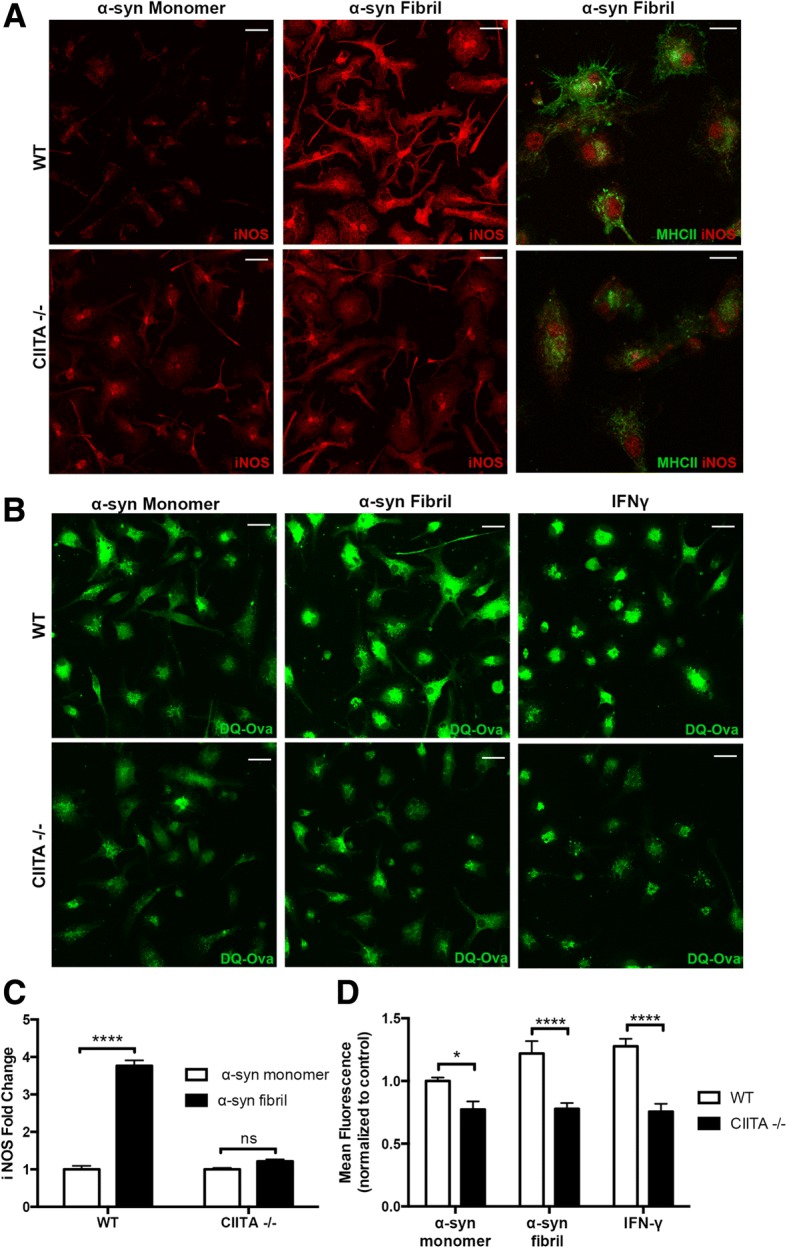
Genetic knock-out of CIITA reduces α-syn-induced inflammation and antigen processing in vitro*.* **a** WT and CIITA −/− primary microglial cultures were treated with α-syn monomer or α-syn fibrils (200 ng/mL) for 2 h and immunolabeled with iNOS (red) and MHCII (green, far right panel). iNOS scale bar represents 25 μm, and MHCII and iNOS image scale bar represents 10 μm. **b** Representative images of WT or CIITA −/− cells treated with DQ-Ovalbumin (green) to assess antigen processing. Cells were treated with α-syn monomer or α-syn fibrils at 200 ng/mL for 2 h, or IFNy (10 ng/mL) as a positive control. Scale bar represents 25 μm. **c** Quantification (fold change over WT microglia α-syn monomer treated control) of iNOS expression in panel **a**. Each group was comprised of 16 individual samples, with four images quantified per sample. Two-way ANOVA with Tukey’s multiple comparisons, ****p < 0.0001. **d** Quantification of DQ-Ovalbumin experiments in **b**. Each experimental group was comprised of eight individual samples, with four images quantified per sample. Samples were normalized to WT microglia α-syn monomer treated control. Two-way ANOVA with Bonferroni’s multiple comparisons, *p < 0.05, ****p < 0.0001

We have previously shown that microglia are able to directly internalize aggregated α-syn, and in response they upregulate MHCII, iNOS, nuclear factor kappa-light-chain-enhancer of activated B cells (NFκB), and release pro-inflammatory cytokines and chemokines [14, 16, 23, 29]. In order to determine whether CIITA expression is essential for the induction of the α-syn-induced pro-inflammatory response, we isolated primary microglia from C57BL/6 J (WT) and CIITA −/− mice on a congenic background and treated cells with 200 ng/mL α-syn fibrils or monomer control for 2 h. 2 h after treatment, iNOS expression measured by ICC was used as a readout for inflammation. WT microglia treated with α-syn fibrils had a mean 2.76-fold increase in iNOS expression via ICC when compared to WT monomer-treated cells (Fig. 1a, c), while in CIITA −/− microglia there was no increase in iNOS expression compared to CIITA −/− monomer treated cells (Fig. 1a, c). We also evaluated MHCII expression as an additional measure of activation, as MHCII is upregulated by activated microglia [14]. In response to α-syn fibril treatment, CIITA −/− microglia had noticeably lower levels of MHCII expression when compared to WT fibril-treated cells via ICC (Fig. 1a).

Since CIITA −/− cells displayed lower MHCII expression in response to α-syn fibril treatment when compared to WT fibril-treated cells (Fig. 1a), we also tested whether knock-out of CIITA attenuates MHCII-dependent antigen processing using an assay based on DQ-Ovalbumin fluorescence. DQ-Ovalbumin is a self-quenching substrate for lysosomal proteases that, upon cleavage in the lysosome, exhibits green fluorescence. To determine whether knockout of CIITA attenuates antigen processing, primary microglia were isolated from WT and CIITA−/− mice and incubated with 200 ng/mL α-syn fibrils, 200 ng/mL monomer, or 10 ng/mL IFNγ. IFNγ was chosen as a positive control that is capable of inducing microglial CIITA activity and MHCII expression through mainly a CIITA promoter I-mediated mechanism (while CIITA promoters III and IV are utilized by lymphoid cells and non-hematopoietic cells) [18, 31]. 2 h after α-syn fibril treatment, cells were washed and incubated with DQ-Ovalbumin for 1 h, fixed, and analyzed. Mean fluorescence was measured for each individual treatment and normalized to WT monomer control. 2 h post-treatment, monomer-treated CIITA −/− microglia had significantly lower DQ-Ovalbumin fluorescence when compared to WT monomer-treated cells, and following α-syn fibril treatment when compared to WT fibril-treated cells (Fig. 1b, d). Importantly, there was no significant induction of antigen processing following α-syn fibril or IFNγ treatment (Fig. 1b, d) in CIITA −/− microglia. These experiments demonstrate that CIITA −/− microglia have a baseline deficit in antigen processing in response to monomer, α-syn fibrils, and IFNγ treatments.

### Knock-out of CIITA attenuates α-syn-induced MHCII expression and prevents neurodegeneration in vivo

**Fig. 2:**
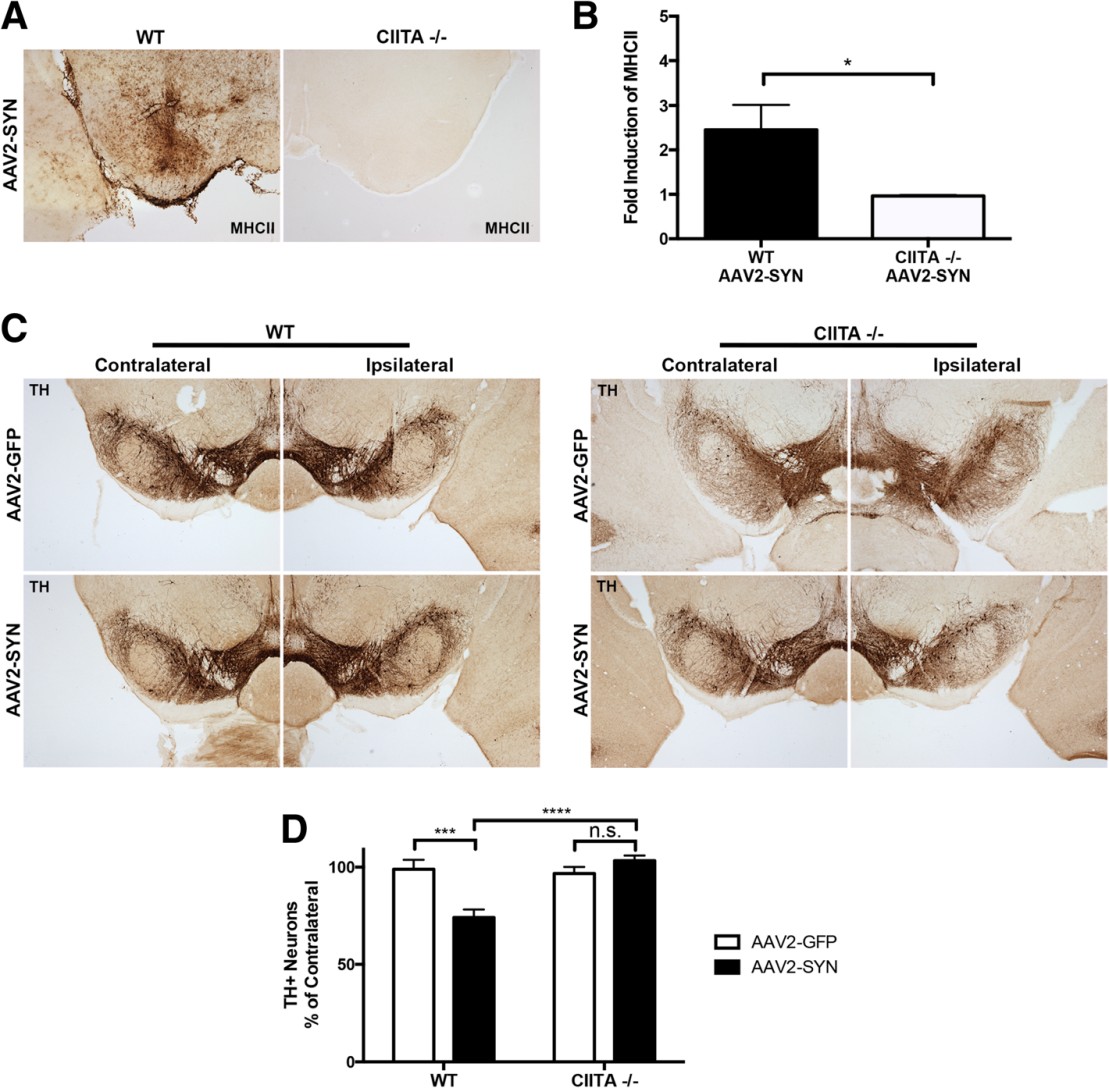
Knock-out of CIITA attenuates α-syn-induced MHCII expression and prevents neurodegeneration in vivo*.* **a** Representative images of AAV2-SYN-induced MHCII expression (DAB, brown) in the ventral midbrain of WT and CIITA −/− mice. Both groups received unilateral AAV2-SYN injection into the ipsilateral SNpc. **b** Quantification of MHCII expression. Sections were analyzed by tiling the entire section and calculating the midbrain mean gray value of both contralateral and ipsilateral hemispheres in ImageJ, adjusting for background. Ipsilateral and contralateral values were used to determine fold induction of MHCII in the ipsilateral side. Data represent the mean MHCII induction, with each data point representing an individual animal. Three brain sections were analyzed per animal, with six animals per group. Equal numbers of male and female mice were used, and an unpaired *t* test was used to analyze data, *p < 0.05. **c** Representative images of TH+ neurons in the substantia nigra. WT and CIITA −/− mice received unilateral injections of AAV2-SYN or AAV2-GFP, and TH neurons were analyzed 6 months later. **d** Quantification of **c**. TH-positive neurons in both the injected (ipsilateral) and uninjected (contralateral) SNpc were counted using unbiased stereology, and numbers are presented as percent of contralateral side. Each group contained 7–9 male and female mice. Equal numbers of males and females were present in each group. Two-way ANOVA with Tukey’s multiple comparisons, ***p < 0.0005, ****p < 0.0001

In order to determine the role of CIITA-dependent antigen processing in response to α-syn over-expression in vivo, we unilaterally injected 2 uL (2.6 × 10^12^ vg/mL) of an adeno-associated virus (AAV2-SYN) into the right SNpc of 8–12-week-old WT or CIITA −/− mice, leading to overexpression of full length human α-syn [15, 24] or green fluorescent protein (GFP) as a control. It has been previously demonstrated that the AAV used in this model selectively transduces neurons [15, 24, 32], and overexpression of α-syn in these neurons results in robust MHCII expression on microglia relative to AAV2-GFP control [14]. 4 weeks post-transduction with AAV2-SYN, the presence of abnormal p-Ser129+ α-syn could be detected in the ipsilateral SNpc of both WT and CIITA −/− mice (Additional file 2A). Additionally, 4 weeks post-transduction with AAV2-SYN, WT mice displayed widespread MHCII expression within the ipsilateral SNpc in parenchymal cells with morphological characteristics of microglia via immunohistochemistry (IHC) as well as in the meninges, whereas CIITA −/− mice had no detectable MHCII expression within the ventral midbrain (Fig. 2a, b).

Since we have previously shown that MHCII expression is required for α-syn-induced inflammation and neurodegeneration in the AAV2-SYN model, we sought to determine whether complete knockout of CIITA is neuroprotective following α-syn expression in vivo. Similar to 4 weeks post-transduction with AAV2-SYN, 6 months post-transduction of AAV2-SYN, the precense of abnormal p-Ser129+ α-syn could still be detected in the ipsilateral SNpc of both WT and CIITA −/− mice (Additional file 2B).To quantify neurodegeneration, 8–10-week-old WT or CIITA −/− mice were unilaterally injected with 2 uL of AAV2-SYN or AAV2-GFP control. 6 months post-transduction, unbiased stereology was performed to quantify TH+ neurons in the SNpc. WT mice transduced with AAV2-SYN revealed a 24.8% loss of tyrosine hydroxylase positive neurons in the ipsilateral SNpc relative to AAV2-GFP control mice, consistent with previous publications [14, 16, 27]. In contrast, knock-out of CIITA was completely neuroprotective against the neurodegenerative effect of AAV2-SYN, as there was no measurable TH+ cell loss by unbiased stereology when compared to AAV2-GFP control (Fig. 2c, d). No differences were observed in the baseline amount of TH+ neurons in the contralateral side between WT and CIITA −/− mice (Additional file 2C). Taken together, these data demonstrate CIITA genetic knock-out attenuates both α-syn-induced MHCII expression and neurodegeneration in vivo.

### Silencing CIITA dampens α-syn mediated myeloid MHCII expression

It has been shown that germline MHCII (and CIITA) knockout mice do not possess functional CD4 T cell populations, as MHCII expression is required for CD4+ T cell maturation in the thymus [17, 18]. This raises the question of whether just MHCII, CD4+ T cells, or both are required for mediating the neurotoxicity observed in response to α-syn expression. In order to bypass any confounding developmental abnormalities and to better investigate the role of CIITA specifically in the midbrain, we packaged six short hairpin RNAs (shRNAs) targeted to CIITA into lentiviruses for delivery directly into the ipsilateral SNpc in vivo. Five lentiviruses were created, targeting different regions of the CIITA gene (Additional file 1A) and characterized in vivo. Lentiviruses (2 uL, titered to 3 × 10^8^ IFU/mL) were delivered concurrently with AAV2-SYN unilaterally into the SNpc of 8–12-week-old WT mice. 4 weeks post-transduction, two lentiviruses (Lentivirus A (LVA), and lentivirus E (LVE)) were selected based on their efficacy in reducing MHCII protein assessed by IHC (Additional file 1B). As an additional measure to ensure that the lentiviruses were not neurotoxic, we performed unbiased stereology on mice transduced with AAV2-GFP, AAV2-SYN, or AAV2-SYN + LGFP (Additional file 1C). The control lentivirus, lentivirus GFP (LGFP) + AAV2-SYN, did not cause any additional loss of TH+ cells compared to AAV2-SYN alone, indicating the lentiviral vector itself was not neurotoxic (Additional file 1C).

**Fig. 3:**
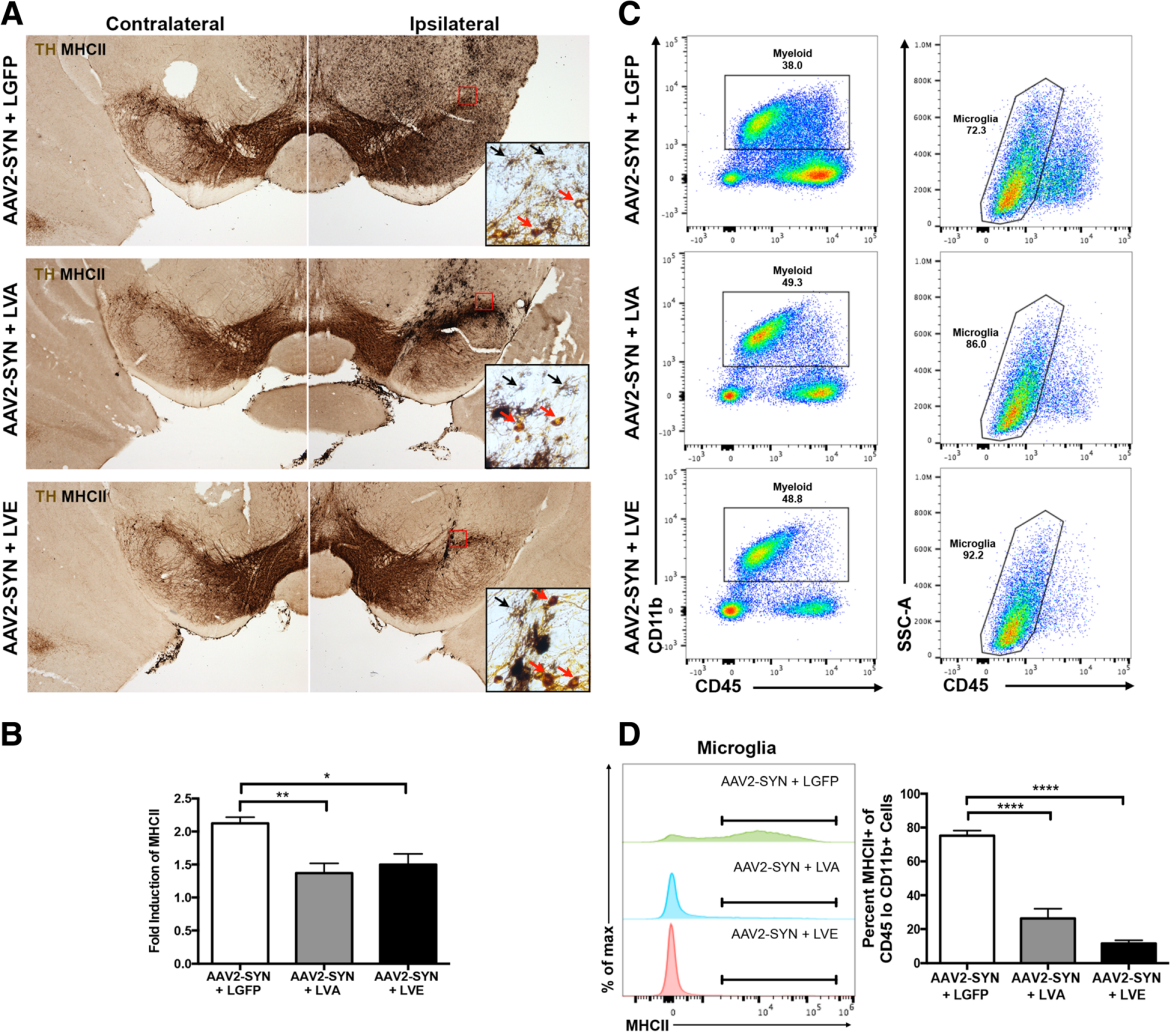
Silencing CIITA dampens α-syn-mediated myeloid MHCII expression. **a** MHCII expression (MHCII, Ni-DAB, black) in the SNpc surrounding dopaminergic neurons (TH, DAB brown) 4 weeks post-transduction in AAV2-SYN + LGFP (control), AAV2-SYN + LVA, and AAV2-SYN + LVE mice. Both contralateral and ipsilateral sides are shown as intra-group controls. Red squares demonstrate location of the × 20 image (inset). Red arrows denote TH+ neurons (DAB), black arrows mark MHCII+ cells (Ni-DAB). **b** Quantification of MHCII induction in AAV2-SYN + LGFP, LVA, or LVE treatment groups, calculated using mean gray value of the ipsilateral and contralateral midbrain in ImageJ, adjusting for background. Ipsilateral and contralateral values were used to determine fold induction of MHCII in the ipsilateral side. Data represent the mean MHCII induction, with each data point representing an individual animal. Three tiled midbrain images were taken per animal, with four to six mice per group. Equal numbers of males and females were used, and data were analyzed by a one-way ANOVA with Dunnet’s multiple comparisons, *p < 0.05, **p < 0.005. **c** Representative flow cytometry plots of mice injected with AAV2-SYN + LGFP, AAV2-SYN + LVA, and AAV2-SYN + LVE at 4 weeks post-transduction. Gates denote MHCII+ microglia. For the flow cytometry experiment, a total of 24 mice were used. Each group consisted of 4 samples, with 2 ventral midbrains pooled per sample. Equal numbers of males and females were used. **d** Representative MFI curves and quantification of MHCII+ microglia, gated as shown in **c**. Gates denoting MHCII+ microglia are shown on MFI curves, and the percent of microglia that are MHCII-positive are graphed. For flow cytometry analysis, the mean ± SEM of samples in each group are plotted. One-way ANOVA with Tukey’s multiple comparisons, ****p < 0.0001

To characterize the effects of local silencing of CIITA on α-syn-induced MHCII expression in more detail, a second group of 8–12-week-old WT mice were unilaterally injected with 2 uL of AAV2-SYN and 2 uL of LGFP control, LVA, or LVE concurrently. 4 weeks post-transduction, MHCII induction was assessed via IHC and quantified (Fig. 3a, Both LVA and LVE significantly attenuated α-syn-induced MHCII expression in the substantia nigra when compared to LGFP treated control animals (Fig. 3a, b). This method of IHC quantification does not discriminate tissue resident microglia from infiltrating monocytes and macrophages, which may also express MHCII at high levels during inflammation. To quantify MHCII expression specifically on microglia using a more sensitive assay, we performed mononuclear cell isolation and flow cytometry on ventral midbrain tissues 4 weeks post-transduction on LGFP control, LVA, and LVE injected mice concurrently transduced with AAV2-SYN bilaterally into the SNpc of WT mice. Microglial populations were defined as CD45^lo^ and CD11b^+^ (Fig. 3c, Additional file 3). In AAV2-SYN + LGFP transduced animals, 75.2% of microglia were MHCII positive 4 weeks post-transduction (Fig. 3d). Both LVA and LVE significantly attenuated microglial MHCII expression in AAV2-SYN-injected mice compared to AAV2-SYN + LGFP (Fig. 3d), as LVA-injected mice had 26.3% MHCII+ microglia and LVE-injected mice had 11.5% positive microglia. Taken together, we show that CNS delivery of these lentiviral siRNAs targeting CIITA decreases α-syn-induced MHCII expression on microglia.

### Silencing CIITA attenuates α-syn-mediated peripheral T cell and monocyte infiltration

**Fig. 4:**
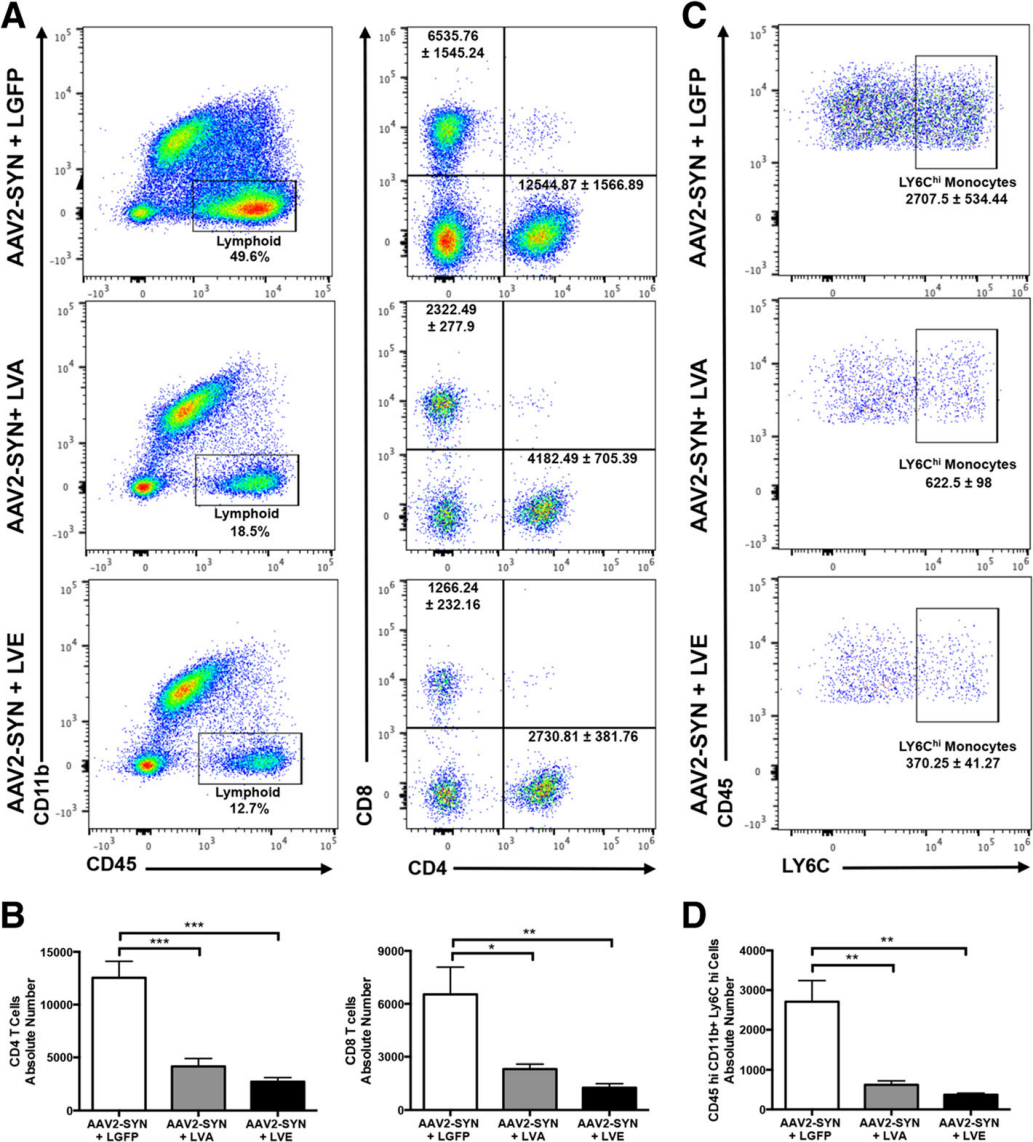
Silencing CIITA attenuates α-syn-mediated T cell and monocyte entry. **a** Representative flow cytometry plots display gating of lymphoid cells (left, representative percent of displayed population is given on plot) followed by CD4 and CD8+ T cells (from the CD45^hi^ CD11b^−^ population, right, mean raw cell number ± SEM is shown on plot) in the ventral midbrains of mice bilaterally injected with AAV2-SYN + LGFP, AAV2-SYN + LVA, or AAV2-SYN + LVE at 4 weeks post-transduction. **b** Quantification of absolute numbers of CD4 and CD8 T cells from **a**. **c** Representative flow cytometry plots showing gating on LY6C^hi^ monocytes (from the CD45^hi^ CD11b^+^ population, mean raw cell number ± SEM is shown on plot) in the ventral midbrains of mice bilaterally injected with AAV2-SYN + LGFP, AAV2-SYN + LVA, or AAV2-SYN + LVE at 4 weeks post-transduction. **d** Quantification of the LY6C^hi^ monocytes from **c**. For **a**–**d**, a total of 24 mice were used—each group consisted of 4 samples with 2 ventral midbrains pooled per sample. Equal numbers of male and female mice were used in these experiments. The mean +/− SEM of samples in each group are plotted. A one-way ANOVA with Tukey’s post hoc test was used for statistical analysis. *p < 0.05, **p < 0.005, ***p < 0.0005

We have previously shown that peripheral immune cell infiltration is a key mechanism of α-syn-induced inflammation and neurodegeneration [15, 27]. In order to determine the role of CIITA-mediated MHCII expression in T cell and peripheral monocyte entry following α-syn overexpression, we injected LVE, LVA, and LGFP control bilaterally into the ipsilateral SNpc concurrent with AAV2-SYN. 4 weeks post-transduction, infiltration of CD4 and CD8 T cells was assessed using mononuclear cell isolation and flow cytometry. T cells were identified as CD45^hi^, CD11b^−^, and gated on CD4 or CD8 (Additional file 3, Fig. 4a). LVA and LVE significantly attenuated numbers of CD4 T cells in the CNS compared to AAV2-SYN + LGFP control (Fig. 4a, b). Additionally, CD8 T cells in the CNS were also significantly reduced at 4 weeks following LVA and LVE transduction. We have previously reported that infiltrating inflammatory monocyte entry in response to α-syn is a key contributor to neurodegeneration [27]. To identify infiltrating monocytes, isolated cells from virally transduced mice were gated on CD45^hi^, CD11b^+^, and Ly6C^hi^ (Additional file 3). The number of infiltrating monocytes (CD11b^+^CD45^hi^Ly6C^hi^) was significantly reduced in AAV2-SYN + LVA- and LVE-treated animals when compared to AAV2-SYN + LGFP control (Fig. 4c, d). These results demonstrate that midbrain targeted silencing of CIITA expression attenuates entry of peripheral CD8 T cells, CD4 T cells, and Ly6C^hi^ monocytes in the ventral midbrain in response to α-syn overexpression.

### Silencing CIITA prevents α-syn-mediated neurodegeneration

**Fig. 5:**
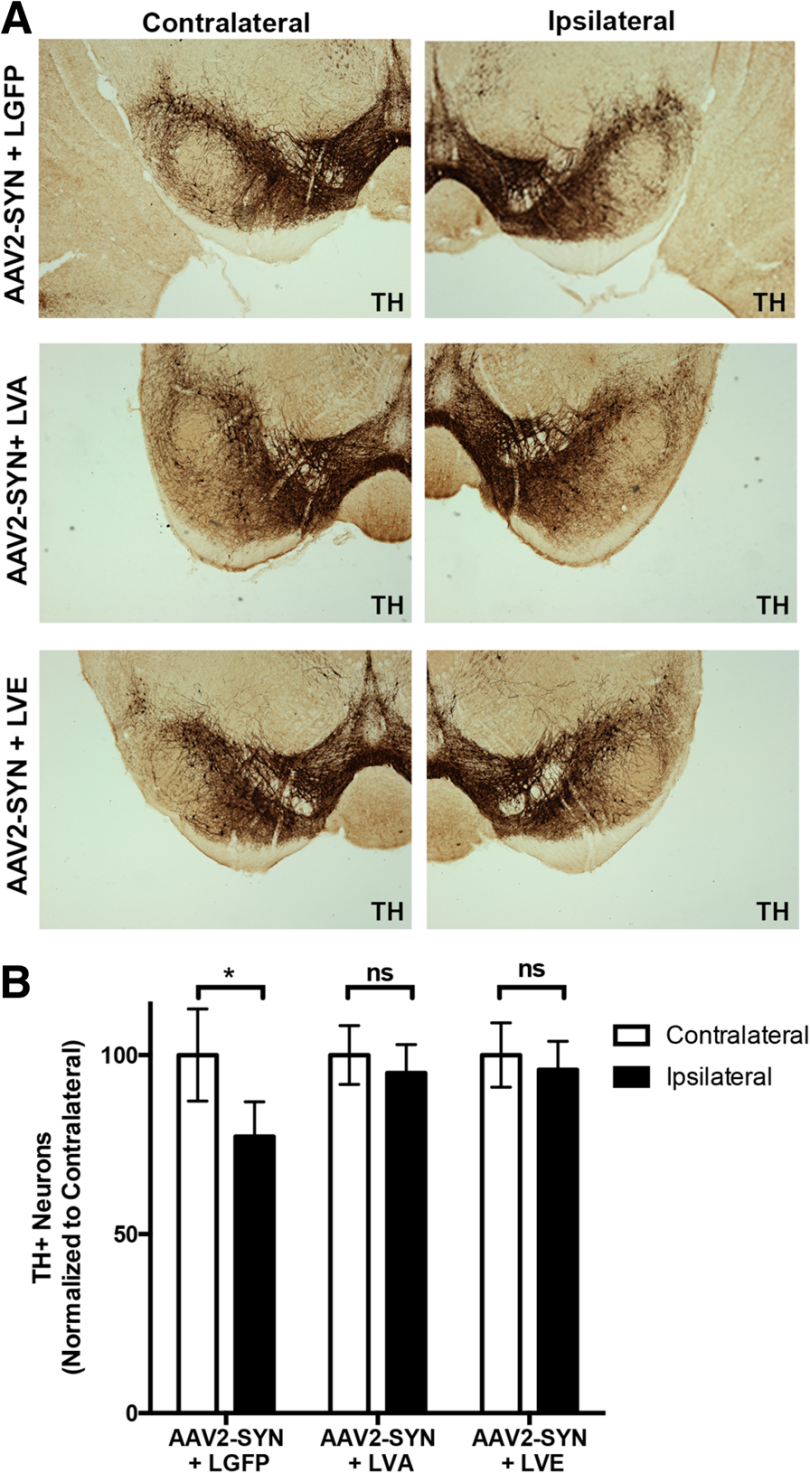
Silencing CIITA prevents α-syn-induced neurodegeneration. **a** Representative × 10 images of TH+ neurons (TH, DAB, brown) in the ipsilateral and contralateral SNpc of WT mice 6 months post-transduction with AAV2-SYN + LGFP, AAV2-SYN + LVA, or AAV2-SYN + LVE. Images were taken with an Olympus BX61 upright microscope. **b** Unbiased stereology counts of TH+ dopaminergic neurons in the SNpc of WT mice 6 months post-transduction with AAV2-SYN + LGFP, AAV2-SYN + LVA, or AAV2-SYN + LVE. Equal numbers of males and females were used. Ipsilateral and contralateral TH neuron counts were normalized to mean of uninjected side for each group. *n* = 8–10 per group. Comparisons were made within each individual treatment group using paired *t* tests and Bonferroni’s multiple comparison correction. *p < 0.016

Finally, we investigated whether local CNS CIITA silencing was neuroprotective in the AAV2 model of PD. We injected AAV2-SYN concurrently with lentiviral constructs unilaterally into the SNpc of 8–10-week-old WT mice. 6 months post-transduction, IHC and unbiased stereology was performed on TH positive neurons in the ipsilateral SNpc using the optical fractionator method. AAV2-SYN + LGFP mice had 22.7% cell loss compared to contralateral side (Fig. 5a, b), matching mice injected with AAV2-SYN alone and in concordance with previously published results [14, 16, 27]. Conversely, the addition of either LVA or LVE with AAV2-SYN was neuroprotective, as no differences were detectable between ipsilateral and contralateral sides (Fig. 5a, b). Additionally, no differences were observed in the baseline amount of TH+ neurons in the contralateral side across the AAV2-SYN + LGFP/LVA/LVE-treated WT mice (Additional file 1D). Therefore, midbrain targeted silencing of CIITA prevents the loss of TH neurons normally observed in the SNpc of AAV2-SYN-injected mice.

## Discussion

Our studies have shown, using both a genetic knockout and a midbrain targeted gene silencing approach, that CIITA expression is required for α-syn-induced inflammation and neurodegeneration in a mouse model of PD. In vitro, CIITA −/− microglia have deficiencies in antigen presentation and the induction of the pro-inflammatory marker iNOS following α-syn fibril treatment. In vivo, CIITA −/− animals have attenuated α-syn-induced MHCII expression in the ventral midbrain and are protected from α-syn-mediated neurodegeneration. We also demonstrated that midbrain targeted silencing of CIITA via lentiviral delivery of shRNA directly into the SNpc reduces α-syn-induced microglial MHCII expression, T cell and monocyte infiltration, and subsequent neurodegeneration. Our results indicate a critical role for CIITA in mediating MHCII expression on microglia in the CNS in response to α-syn and that CIITA is required for α-syn-mediated inflammation and neurodegeneration in a mouse model of PD.

To model α-syn-mediated inflammation and neurodegeneration in vivo, we selected the AAV2-SYN overexpression model of PD. This model allows for targeted overexpression of full-length human α-syn in neurons of the substantia nigra of mice [24]. This expression of α-syn results in abnormal α-syn (p-Ser129+), inflammation, and subsequent neurodegeneration 6 months post transduction [14, 16, 27]. It is important to consider caveats of this model which include the need for surgical injection of the virus and induction of a high level of α-syn expression, but it does model many of the features observed in human PD [33, 34]. The α-syn expression, inflammation, and neurodegeneration in this model have been thoroughly characterized and are reproducible [14–16, 27–29]. Using this AAV model, we have previously reported microglial MHCII expression, T cell entry, monocyte entry, and loss of dopaminergic neurons in the SNpc in this model [14, 16, 27]. These inflammatory components in the AAV2-SYN model mirror what can be observed in human PD [6–8, 35] as well as the hallmark dopaminergic cell loss and p-Ser129+ α-syn inclusions.

Using the AAV2-SYN model and primary microglia cultures, we used a genetic knockout approach to test the hypothesis that CIITA is critical for the induction and function of microglial MHCII protein in response to abnormal α-syn. When treated with α-syn pre-formed fibrils, CIITA knock-out microglia displayed less MHCII expression, DQ-Ovalbumin processing, and iNOS expression in vitro than WT microglia (Fig. 1), indicating that CIITA is crucial for microglial MHCII induction and downstream effector function. In vitro, we did observe higher baseline expression levels of both iNOS and MHCII in CIITA −/− microglia, a result that is different from our in vivo observations of MHCII expression in CIITA deficient animals (Fig. 2). It is important to note that there are other minor transcriptional pathways that can lead to MHCII expression [36]. Additionally, a number of papers have reported fundamental differences in the genetic profiles of cultured primary microglia compared to their freshly isolated adult counterparts [37] indicating that in vitro, microglia may not achieve a full resting state. Furthermore, it is important to note that microglia in a healthy mouse brain have very low expression of MHCII at baseline [14, 38, 39]. Therefore, it is difficult to know whether the in vitro expression at baseline is due to compensatory transcriptional pathways, intrinsic differences between in vitro and in vivo cells, or baseline biological differences in activation status between WT and CIITA knock-out. Importantly, though, CIITA −/− cells had deficits in upregulation of MHCII and iNOS despite the potentially higher baseline expression compared to WT cells. For these reasons, we decided to substantiate our findings in vivo. The attenuated effect on MHCII induction observed in CIITA −/− mice was even more drastic than that found in vitro, as knock-out mice (Fig. 2) had no sign of ventral midbrain MHCII expression in response to AAV2-SYN at 4 weeks post-transduction. Additionally, CIITA −/− mice were protected from the TH^+^ neurodegeneration observed in WT mice 6 months post AAV2-SYN transduction (Fig. 2). It has been shown in multiple cell types, including splenic B cells and macrophages, that CIITA is required for MHCII upregulation upon immune challenge [18, 40], and our global CIITA knock-out results confirm that CIITA is also required for α-syn-mediated microglial MHCII induction in vitro and in vivo*.* Additionally, our findings support the idea that CIITA is a viable target to modulate MHCII expression in the CNS as a potential therapeutic target in PD.

We have previously demonstrated the importance of MHCII in the inflammatory signaling pathways leading to neurodegeneration in the AAV2-SYN model [14]. A caveat to those findings that required further investigation is the fact that germline MHCII or CIITA knockout mice do not possess functional CD4 T cells, as MHCII expression is required for CD4 T cell maturation in the thymus [17, 18]. This raises the question of whether just MHCII, CD4 T cells, or both are responsible in mediating the neurotoxicity observed in response to α-syn expression. In order to better address this question as well as to avoid any other confounding developmental factors of a genetic knockout, we employed the use of lentivirus delivered shRNAs targeting CIITA and subsequent MHCII expression directly into the CNS. We chose lentiviral delivery for multiple reasons: it is a reliable and effective way to deliver siRNAs into the CNS [41], they provide prolonged expression of packaged proteins [42], and we required infectivity of antigen presenting cells in the CNS [42]. To that end, the use of our lentiviruses to silence CIITA expression allows for a CNS-specific approach that preserves the development of the peripheral immune system, notably CD4 T cell populations. Using this midbrain selective CIITA silencing method, we showed that LVA or LVE + SYN-treated mice display less microglial MHCII expression (Fig. 3) as well as TH^+^ neuroprotection (Fig. 5) compared to AAV2-SYN + LGFP-treated control mice. These data confirm the critical role of CIITA in modulating microglial MHCII expression and the subsequent inflammatory and neurodegenerative response to α-syn overexpression. MHCII and infiltration of inflammatory cells was used as a readout for inflammation, although it is also likely that alterations in CIITA expression affects other markers of activation and readouts of microglial effector function, such as morphology and cytokine release [43]. It is important to note that the CIITA silencing paradigm used in our experiments may also affect the CIITA/MHCII expression of non-microglial cells such as astrocytes and neurons [31, 44–46]. Further investigation is needed to determine if the reported non-canonical MHCII expression on these non-microglial CNS cells contributes to the neurotoxicity or peripheral immune cell recruitment seen in response to α-syn overexpression. However, our data suggests that the induction of MHCII+ microglia in resonse to AAV2-SYN is an initiator and/or driver of the disease process in this mouse model of PD.

We have shown that a downstream effect of blocking microglial MHCII expression through CIITA targeting is a significant reduction in T cell and monocyte infiltration into the midbrain (Fig. 4). The reduction of both T cells and monocytes is important as both have been implicated in the PD disease pathogenesis. Regarding the involvement of T cells in PD, increased numbers of CD4 and CD8 T cells can be found in PD postmortem brain [8]. Recently, the presence of α-syn auto-reactive CD4 and CD8 T cells has been described in PD patient blood [13]. Moreover, previous work in the MPTP model of PD has shown that CD4 T cells play an essential role in neurodegeneration [8, 47, 48]. Additionally, the finding that SNPs in the HLA region, part of the MHCII complex that is closely associated with a CD4 T cell response, are associated with greater PD risk [10–12] has stressed that CD4 T cells play a role in establishing the disease state. However, it is important to note that there is evidence that shows direct CD8 T cell-mediated DA neuron degeneration [49] and that some PD-related genes are involved in CD8 T cell activation [50].

Less is known about the contribution of monocytes to PD, but an enriched subset of pro-inflammatory monocytes has been observed in PD patient blood [35]. More generally, blood-derived monocytes have been shown to infiltrate tissues including the CNS during active disease states where they can promote the destruction of CNS tissue as is the case in experimental autoimmune encephalomyelitis (EAE, a mouse model of multiple sclerosis) [51]. Additionally, we have shown that blocking monocyte entry is neuroprotective in the AAV2-SYN model [27]. Taken together, it may be that in PD, recognition by T cells of abnormal forms of α-syn presented on microglia MHCII may trigger aberrant cytokine production and disease producing effector functions that includes the recruitment of peripheral monocytes that lead to the observed CNS dysfunction [52, 53]. It is important to remember that this study did not employ any fate mapping paradigm to track the peripheral immune infiltrate in response to AAV2-SYN so it is unclear if CIITA/MHCII signaling is more important for the entry of peripheral immune cells and/or the engraftment of such immune cells into the CNS. Future studies are needed to better understand the connections between CIITA-mediated MHCII upregulation, T cell entry, and monocyte entry in response to α-syn.

Current treatments for PD serve only to treat the symptoms that are a consequence of dopamine deletion but do little to halt or alter disease progression. The development of disease modifying therapies to help reverse, slow down, or prevent the onset of PD pathogenesis is greatly needed. One potential avenue for the development of these therapies would be the use of immunomodulatory strategies, as they have been successful in other neurodegenerative and autoimmune disorders. Our data presented here in a mouse model of PD points to the critical role of CIITA in contributing to the MHCII-mediated inflammation and subsequent neurodegeneration observed in the model. Our studies in an α-syn model of PD show the feasibility and effectiveness of targeting CIITA expression to attenuate α-syn-induced microglial MHCII expression, peripheral T cell and monocyte infiltration, and subsequent neurodegeneration without compromising the development of the peripheral immune system. Future studies are warranted to determine whether targeting CIITA in human disease is a viable disease modifying therapeutic.

## Conclusions

In summary, our data provide evidence that CIITA is required for α-syn-induced MHCII expression and subsequent infiltration of peripheral immune cells in to the midbrain. Additionally, we demonstrate that knock-out or silencing of CIITA expression in the midbrain prevents neurodegeneration. These data provide further support that the disruption or modulation of antigen processing and presentation via targeting CIITA is a promising approach for therapeutic development.

## Additional files

Additional file 1: Generation and selection of lentiviral constructs. (A) Sequences of shRNAs targeted to CIITA and packaged into lentiviral constructs. Each lentivirus was titered to 3 × 10^8^ IFU/mL. (B) Mice were injected with AAV2-SYN and each lentivirus into the SNpc. 4 weeks post-transduction, MHCII staining was quantified by a blind rater score. 5–6 mice were used per group, and median value is plotted. Individual points represent single animals. (C) AAV2-GFP, AAV2-SYN, or AAV2-SYN + LGFP was injected into the SNpc of mice. 6 months post transduction, TH+ neurons were quantified using unbiased stereology at plotted as a % of the contralateral side. Each group contained 7–10 mice and equal numbers of male and female mice were used. Mean ± SEM is plotted. One-way ANOVA with Tukey’s multiple comparisons, **p < 0.005. ns = not significant. (D) Quantification of TH-positive neurons in the uninjected (contralateral) SNpc of AAV2-SYN + LGFP/LVA/LVE-treated WT mice 6 months post viral transduction. As in Fig. 5b, counts were obtained using unbiased stereology and total numbers are reported. For each group, equal numbers of males and females were used, *n* = 8–10 per group. One-way ANOVA, ns = not significant. (PDF 369 kb)

Additional file 2: WT and CIITA −/− expression of alpha-synuclein and baseline SNpc dopaminergic neuron counts. (A) Immunolabeling of p-Ser129 in WT and CIITA −/− mice at 4 weeks post-transduction with AAV2-SYN. Contralateral, uninjected side is shown as a control. Black boxes demonstrate × 20 zoom images, and red boxes indicate the location of zoom image. (B) Immunolabeling of p-Ser129 of WT and CIITA −/− mice at 6 months post-transduction. Black boxes demonstrate × 20 zoom images, and red boxes indicated location of zoom image. (C) Quantification of TH positive neurons in the uninjected (contralateral) SNpc of AAV2-GFP/SYN treated WT and CIITA −/− mice 6 months post viral transduction. As in Fig. 2d, counts were obtained using unbiased stereology and total numbers are reported. For each group, equal number of males and females were used, *n* = 7–9 per group. One-way ANOVA, ns = not significant. (PDF 4278 kb)

Additional file 3: Gating strategy for flow cytometric analysis. Single cell suspensions were stained for surface markers, examined on an Attune Nxt flow cytometer, and analyzed using FlowJo software. Cells were gated on single, live cells before being gated into specific leukocyte populations. (PDF 1461 kb)

## Abbreviations

AAV: Adeno-associated virus
ANOVA: Analysis of variance
APC: Antigen presenting cell
CCR2: C-C chemokine receptor type 2
CIITA: Class II transactivator
CNS: Central nervous system
DAB: Diaminobenzadine
EAE: Experimental autoimmune encephalomyelitis
GWAS: Genome-wide association studies
HLA: Human leukocyte antigen
ICC: Immunocytochemistry
IHC: Immunohistochemistry
iNOS: Inducible nitric oxide synthase
MHCII: Major histocompatibility complex II
PD: Parkinson’s disease
p-ser129: Phospho-serine 129
SEM: Standard error of mean
shRNA: Short hairpin ribonucleic acid
SNpc: Substantia nigra pars compacta
TBS: Tris-buffered saline
α-syn: α-synuclein
