# Supplementary material for: Targeting of the class II transactivator attenuates inflammation and neurodegeneration in an alpha-synuclein model of Parkinson’s disease
Source: J Neuroinflammation. 2018 Aug 30;15:244. doi: 10.1186/s12974-018-1286-2 (PMC6117927; doi:10.1186/s12974-018-1286-2)

A

LVA: AAGTGTACAAGGTAGCTGG

LVC: TGTCTTTGATTTCATTGCA

LVD: TATTGTACAAGCTCAGCCT

LVE: TGTATCACTCAAGGAGGCC

LVF: TTGTSTCACTCAAGGAGGC

3 x 10[8] IFU/mL

B

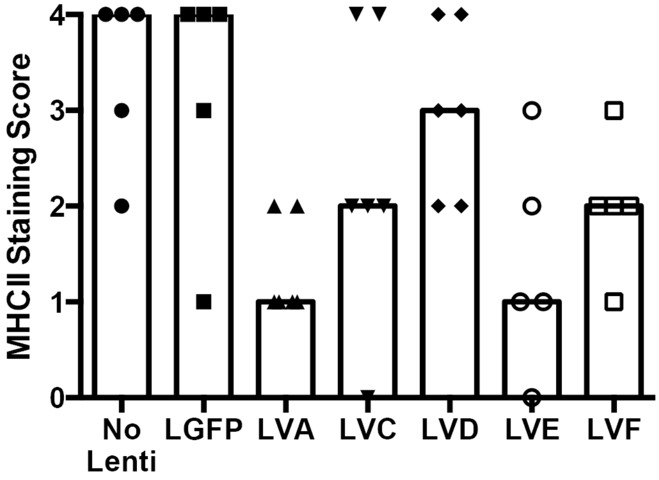

C

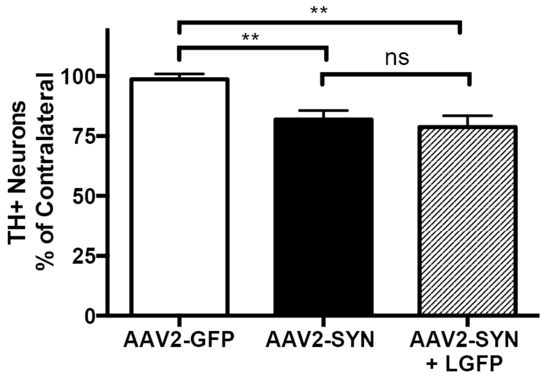

D

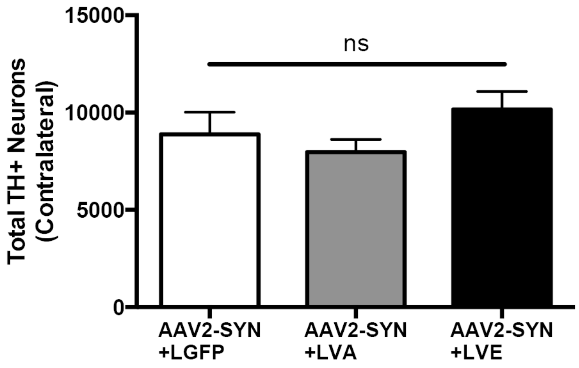

Supplement: Supplementary file 1 — Generation and selection of lentiviral constructs. (A) Sequences of shRNAs targeted to CIITA and packaged into lentiviral constructs. Each lentivirus was titered to 3 × 10^8^ IFU/mL. (B) Mice were injected with AAV2-SYN and each lentivirus into the SNpc. 4 weeks post-transduction, MHCII staining was quantified by a blind rater score. 5–6 mice were used per group, and median value is plotted. Individual points represent single animals. (C) AAV2-GFP, AAV2-SYN, or AAV2-SYN + LGFP was injected into the SNpc of mice. 6 months post transduction, TH+ neurons were quantified using unbiased stereology at plotted as a % of the contralateral side. Each group contained 7–10 mice and equal numbers of male and female mice were used. Mean ± SEM is plotted. One-way ANOVA with Tukey’s multiple comparisons, **p < 0.005. ns = not significant. (D) Quantification of TH-positive neurons in the uninjected (contralateral) SNpc of AAV2-SYN + LGFP/LVA/LVE-treated WT mice 6 months post viral transduction. As in Fig. 5b, counts were obtained using unbiased stereology and total numbers are reported. For each group, equal numbers of males and females were used, n = 8–10 per group. One-way ANOVA, ns = not significant. (PDF 369 kb) [file 12974_2018_1286_MOESM1_ESM.pdf]
