# Supplementary figures and images for: Targeting of the class II transactivator attenuates inflammation and neurodegeneration in an alpha-synuclein model of Parkinson’s disease
Source: J Neuroinflammation. 2018 Aug 30;15:244. doi: 10.1186/s12974-018-1286-2 (PMC6117927; doi:10.1186/s12974-018-1286-2)

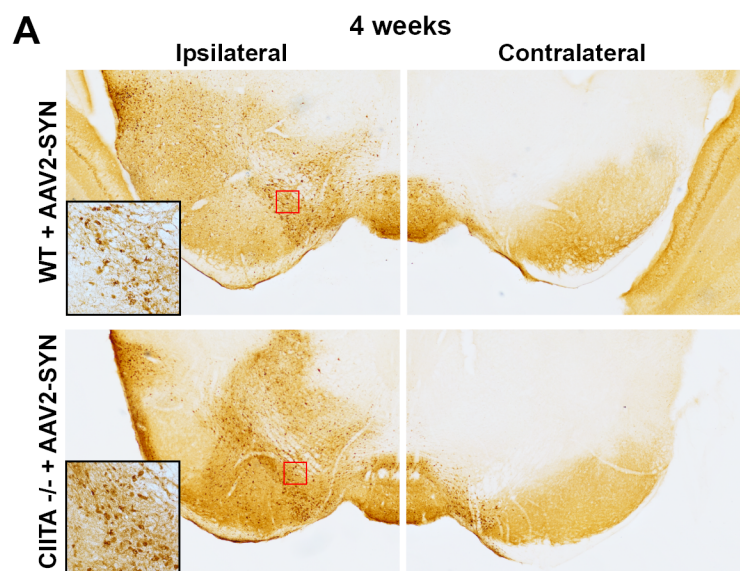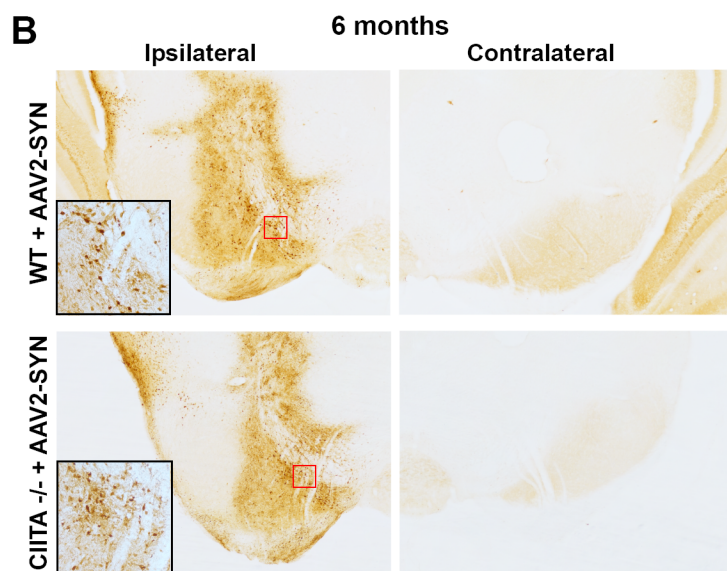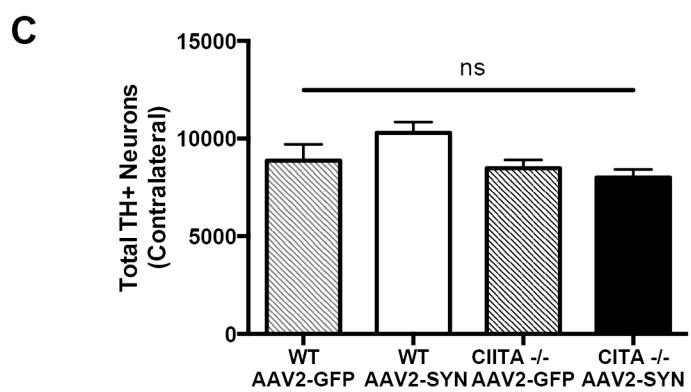

Supplement: Supplementary file 2 — WT and CIITA −/− expression of alpha-synuclein and baseline SNpc dopaminergic neuron counts. (A) Immunolabeling of p-Ser129 in WT and CIITA −/− mice at 4 weeks post-transduction with AAV2-SYN. Contralateral, uninjected side is shown as a control. Black boxes demonstrate × 20 zoom images, and red boxes indicate the location of zoom image. (B) Immunolabeling of p-Ser129 of WT and CIITA −/− mice at 6 months post-transduction. Black boxes demonstrate × 20 zoom images, and red boxes indicated location of zoom image. (C) Quantification of TH positive neurons in the uninjected (contralateral) SNpc of AAV2-GFP/SYN treated WT and CIITA −/− mice 6 months post viral transduction. As in Fig. 2d, counts were obtained using unbiased stereology and total numbers are reported. For each group, equal number of males and females were used, n = 7–9 per group. One-way ANOVA, ns = not significant. (PDF 4278 kb) [file 12974_2018_1286_MOESM2_ESM.pdf]

**A**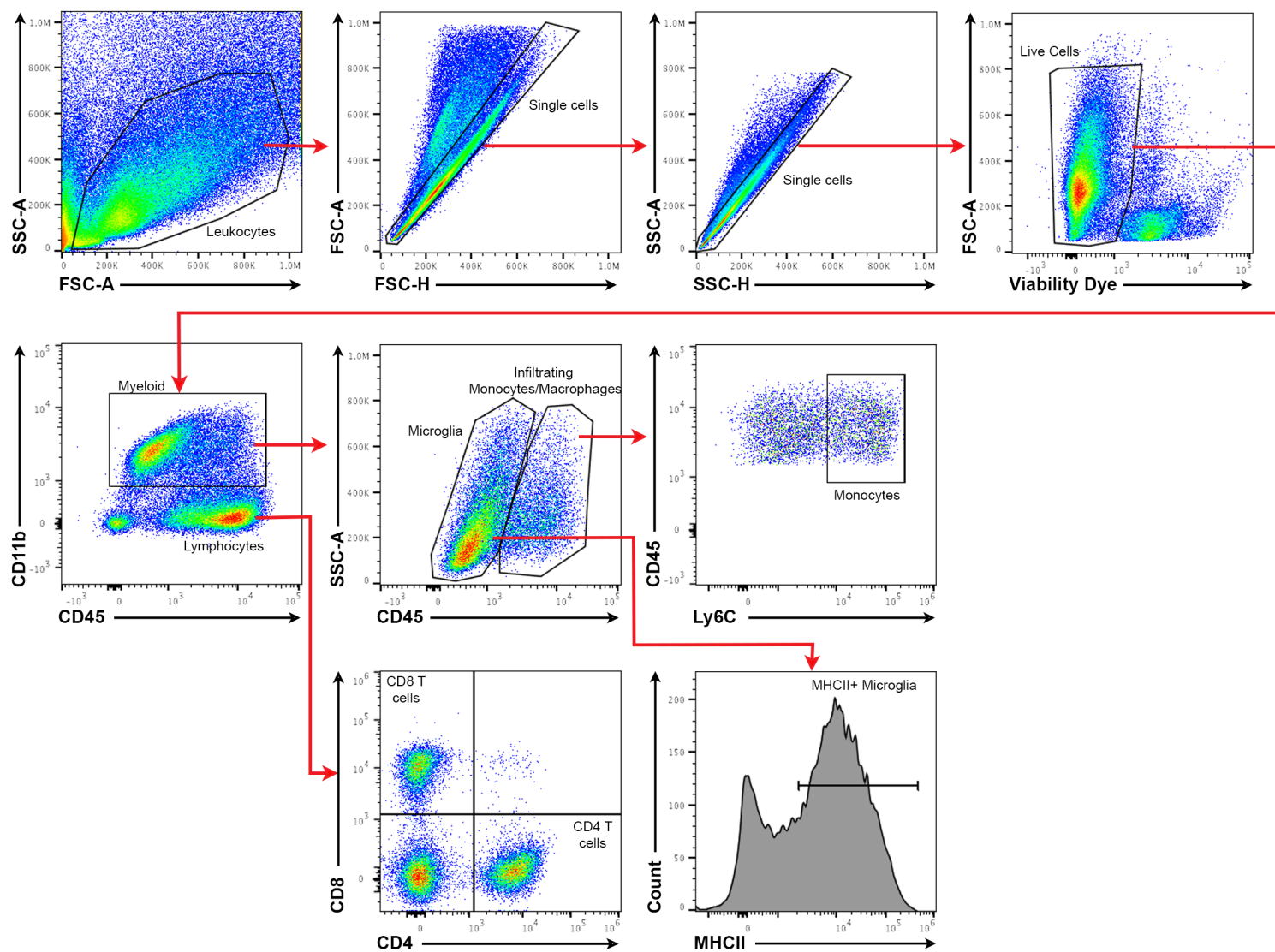

Supplement: Supplementary file 3 — Gating strategy for flow cytometric analysis. Single cell suspensions were stained for surface markers, examined on an Attune Nxt flow cytometer, and analyzed using FlowJo software. Cells were gated on single, live cells before being gated into specific leukocyte populations. (PDF 1461 kb) [file 12974_2018_1286_MOESM3_ESM.pdf]
